# Serum Proteomic Profiling Uncovers LGALS3BP as a Potential Biomarker for Idiopathic Pulmonary Arterial Hypertension

**DOI:** 10.1111/crj.70138

**Published:** 2025-11-27

**Authors:** Mingfei Li, Qi Jin, Wenzhi Pan, Dan Tian, Peng Wang, Dandan Chen, Yuan Zhang, Shasha Chen, Daxin Zhou, Lihua Guan, Junbo Ge

**Affiliations:** ^1^ Department of Cardiology, Zhongshan Hospital, Fudan University Shanghai Institute of Cardiovascular Diseases Shanghai China; ^2^ State key Laboratory of Cardiovascular Diseases, Zhongshan Hospital Fudan University Shanghai China; ^3^ National Clinical Research Center for Interventional Medicine Shanghai China; ^4^ Department of Pharmacy, Zhongshan Hospital Fudan University Shanghai China

**Keywords:** idiopathic pulmonary arterial hypertension, LGALS3BP, proteomics, serum biomarker

## Abstract

**Background:**

Idiopathic pulmonary arterial hypertension (IPAH) is a progressive and fatal disease characterized by pathological pulmonary vascular remodeling and right heart failure. Current biomarkers for IPAH demonstrate limited clinical utility, necessitating the discovery of noninvasive serum biomarkers to facilitate early diagnosis and prognosis.

**Objective:**

To identify novel serum biomarkers for IPAH using tandem mass tag–based quantitative proteomics and validate their clinical relevance.

**Methods:**

Serum samples from five IPAH patients and five age/sex‐matched healthy controls (discovery cohort) were analyzed by tandem mass tag proteomics to screen differentially expressed proteins (DEPs). Functional enrichment and protein–protein interaction network analyses were performed. Serum galectin‐3 binding protein (LGALS3BP) levels were validated by enzyme‐linked immunosorbent assay (ELISA) in an expanded cohort (30 IPAH patients vs. 30 controls).

**Results:**

Proteomic analysis identified 401 proteins and 1554 peptide fragments, with 114 DEPs between groups (47 up‐regulated and 67 down‐regulated). In biological processes, DEPs are primarily enriched in adaptive immune response, followed by signal transduction. Extracellular exosome and extracellular region are the most commonly enriched cell components. For molecular function, DEPs are mainly involved in antigen binding and calcium ion binding, with the top 30 Gene Ontology terms exhibiting similar distribution patterns between up‐ and down‐regulated DEPs. Pathway analysis revealed significant enrichment in complement/coagulation cascades, immune response, and extracellular matrix remodeling. LGALS3BP was observed to be significantly up‐regulated in IPAH serum compared with controls (fold change = 1.36, *p* < 0.001), a finding validated by independent ELISA (IPAH: 6.43 ± 1.73 μg/mL vs. controls: 2.33 ± 1.06 μg/mL; *p* < 0.001).

**Conclusions:**

Integrated proteomic profiling and clinical validation provide the first evidence of elevated serum LGALS3BP in IPAH, indicating its putative role in pathogenesis and translational medicine.

## Graphical abstract

Serum proteomic analysis revealed 114 differentially expressed proteins in IPAH. LGALS3BP, validated as elevated by ELISA, emerges as a new biomarker for pathogenesis and translation.

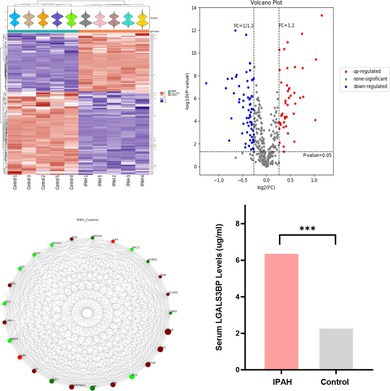

## 1 Introduction

Idiopathic pulmonary arterial hypertension (IPAH) is a rare and progressive disorder with an estimated prevalence of 15 cases per million, characterized by pathological pulmonary vascular remodeling and elevated mean pulmonary arterial pressure (mPAP) [1]. Disease progression is defined by a sustained increase in pulmonary vascular resistance (PVR), culminating in right ventricular dysfunction and eventual failure, which represents the cause of mortality in this population [2, 3]. Despite advancements in diagnostic modalities and therapeutic strategies, contemporary therapeutic interventions mainly focus on pulmonary vasodilation rather than the underlying vascular pathology, accounting for persistently high long‐term mortality rates [4, 5].

The insidious nature of IPAH, coupled with the absence of pathognomonic early symptoms, necessitates invasive right heart catheterization (RHC) for definitive diagnosis. While N‐terminal brain natriuretic peptide precursor (NT‐proBNP) serves as a conventional biomarker for assessing right ventricular function, its diagnostic accuracy is confounded by comorbidities such as left heart disease and renal impairment [6, 7]. Given the compromised clinical status of IPAH patients and relative contraindications associated with lung biopsies, considerable interest has emerged in identifying noninvasive serum biomarkers capable of elucidating disease mechanisms and enabling early detection.

Tandem mass tag (TMT)–based quantitative proteomics has emerged as a powerful tool for high‐throughput biomarker discovery, allowing simultaneous quantification of protein expression across multiple samples. Potential biomarkers may serve diagnostic, prognostic, or therapeutic roles, including disease stratification, outcome prediction, treatment response monitoring, and identification of novel therapeutic targets [8]. In this study, we applied TMT proteomics to identify differentially expressed proteins (DEPs) in the serum of IPAH patients compared with healthy controls, followed by rigorous validation of potential serum biomarkers to ensure reproducibility and scientific robustness.

## 2 Methods

### 2.1 Patients Enrollment

For the discovery‐phase proteomic analysis, we prospectively enrolled five consecutive treatment‐naïve adult patients newly diagnosed with IPAH in our center between January and December 2018. The diagnosis of IPAH was established in accordance with the 2015 European Society of Cardiology and the European Respiratory Society Guidelines for the Diagnosis and Treatment of Pulmonary Hypertension, and confirmed by RHC demonstrating a mPAP ≥ 25 mmHg at rest, pulmonary capillary wedge pressure (PCWP) ≤ 15 mmHg, and PVR > 3 Wood units, after exclusion of other etiologies [9]. Exclusion criteria comprised (1) prior exposure to PAH‐specific therapies (including endothelin receptor antagonists, phosphodiesterase‐5 inhibitors, or prostacyclin pathway agents) and (2) significant comorbidities potentially confounding proteomic analyses, such as uncontrolled diabetes mellitus (HbA1c > 8%), chronic kidney disease (eGFR < 60 mL/min/1.73 m^2^), or malignancy. To ensure comparability and minimize potential confounding, five age‐, sex‐, and body mass index‐matched healthy volunteers were recruited from individuals undergoing routine health examinations at our hospital during the same period. These controls had no clinical evidence of cardiopulmonary disease, hypertension, diabetes, or renal dysfunction. For the validation phase, a separate cohort comprising 30 IPAH patients and 30 contemporaneous healthy controls was selected between January 2019 and December 2019. Controls were similarly matched to patients by sex and body mass index and were confirmed free of significant cardiopulmonary or systemic conditions. Comprehensive baseline characterization including detailed demographic profiling, laboratory assay, and auxiliary examination were recorded. This study was approved by the Zhongshan Hospital Institutional Ethics Committee (Approval Number: B2021‐383R). Written informed consent was obtained from patients before the study.

### 2.2 RHC

All RHC procedures were performed under fluoroscopic guidance in a standardized cardiac catheterization laboratory at our center. Following local anesthesia with 1% lidocaine, percutaneous access was established via the left subclavian vein, femoral vein, or internal jugular vein using the Seldinger technique, with subsequent placement of a 6‐French introducer sheath. Intravenous heparin was administered for systemic anticoagulation to avoid catheter‐associated thrombosis. Baseline preprocedural parameters, including noninvasive blood pressure and room air fingertip oxygen saturation, were routinely recorded without supplemental oxygen. Hemodynamic assessment was conducted using a Swan–Ganz catheter (Edwards Lifesciences) connected to a dedicated monitoring system (Vigilance II monitor, Edwards Lifesciences). Pressure waveforms and oxygen saturation measurements were sequentially obtained from (1) superior and inferior vena cava, (2) right atrium, (3) right ventricle (peak systolic and end‐diastolic right ventricle pressure), and (4) pulmonary artery (systolic, diastolic, mPAP, and mean PCWP). Cardiac output was determined by the thermodilution technique using three serial injections of 10‐mL ice‐cold 0.9% saline, with < 10% variation between measurements required for acceptance. Cardiac index was derived by indexing cardiac output to body surface area, while PVR was calculated as (mPAP − PCWP)/cardiac output [10].

### 2.3 Sample Collection and Measurement

Peripheral venous blood samples (10 mL) were collected after an overnight fast using standard venipuncture technique and immediately placed on ice. Within 30 min of collection, samples were centrifuged at 4°C (3000 rpm for 10 min) to obtain the serum in the cardiac laboratory. Galectin‐3 binding protein (LGALS3BP), also known as Gal‐3BP, was quantified in duplicate using a validated commercial enzyme‐linked immunosorbent assay (ELISA) kit (Human Galectin‐3BP Quantikine ELISA, R&D Systems) following manufacturer protocols. Optical density readings were obtained using a microplate reader (Denley Dragon Wellscan Mk3 Instrument, Thermo Fisher). Sample concentrations were interpolated from the standard curve with a coefficient of variation < 15% between runs. All measurements were performed by technicians blinded to clinical data.

### 2.4 Proteomic Analysis by TMT Labeling

Serum samples from IPAH patients and matched controls were subjected to quantitative proteomic profiling using TMT 10‐plex labeling (Thermo Fisher Scientific; 10 labels are 126, 127N, 127C, 128N, 128C, 129N, 129C, 130N, 130C, and 131 Da). Briefly, proteins were extracted from serum, reduced, alkylated, and digested with trypsin. The resulting peptides were labeled with TMT reagents according to the manufacturer's protocol, with one channel reserved for a pooled reference sample for cross‐run normalization.

After quenching the labeling reaction, TMT‐labeled peptides were pooled, desalted using C18 solid‐phase extraction, and fractionated by high‐pH reversed‐phase chromatography to reduce complexity. Fractions were analyzed by nanoLC‐MS/MS on an Orbitrap Fusion Lumos mass spectrometer (Thermo Fisher Scientific) coupled to an EASY‐nLC 1200 system. Peptides were separated on a C18 column (75 μm × 15 cm) with a 60‐min gradient (5%–35% acetonitrile/0.1% formic acid) and analyzed in data‐dependent acquisition mode. MS1 spectra were acquired at 120 000 resolution (*m/z* 350–1500), followed by higher energy collisional dissociation fragmentation (normalized collision energy = 32) of the top 10 precursors for MS2 quantification at 45,000 resolution. Raw data were processed using Proteome Discoverer (v2.4) against the UniProt human database. Search parameters included a 1% false discovery rate (FDR) threshold. TMT reporter ions were quantified with a minimum of two unique peptides per protein.

### 2.5 Differential Protein Analysis

DEPs were identified using fold change (FC) and *p* values (calculated from the Student's *t* test). FC represented expression differences between IPAH and control groups, with predefined thresholds of |FC| ≥ 1.2 and *p* < 0.05 for DEP selection. For quality control, unsupervised hierarchical clustering (R language and Pearson correlation) was performed to visualize DEP patterns in heatmaps.

### 2.6 Functional Enrichment and Network Analysis

DEPs were functionally annotated using Gene Ontology (GO, https://www.geneontology.org/) terms and Kyoto Encyclopedia of Genes and Genomes (KEGG, https://www.genome.jp/kegg/) pathways. Enrichment significance was evaluated via hypergeometric testing, with *p* values adjusted for multiple comparisons using the Benjamini–Hochberg method (FDR < 0.05). Protein–protein interaction (PPI) networks were constructed using the STRING database (https://string‐db.org/). Network analysis was performed using Python's NetworkX package to identify and visualize the 25 most interconnected nodes within the protein interaction network.

### 2.7 Statistical Analysis

Statistical analyses were performed using SPSS 26.0 (IBM Corp., Armonk, NY, USA) for data processing and computational statistics. Data visualization was conducted using GraphPad Prism 8 (GraphPad Software, San Diego, California, USA). Normally distributed continuous variables were expressed as mean ± standard deviation. A two‐tailed *p* value < 0.05 was defined as statistically significant for all comparative analyses. For proteomic data processing, Proteome Discoverer 2.4 (Thermo Fisher Scientific) was employed with the following stringent filtering criteria: proteins required at least one unique peptide (≥ 1.0) and a Sequest HT score > 0 to ensure analytical reliability. Proteins failing to meet these quality control thresholds (score = 0) were systematically excluded from subsequent analyses to maintain data integrity.

## 3 Results

### 3.1 Baseline Characteristics

In the discovery‐phase cohort, the IPAH patient group (*n* = 5) and healthy controls (*n* = 5) exhibited well‐matched demographic characteristics (Table S1), with no statistically significant differences in age (38.9 ± 11.3 vs. 37.8 ± 12.2 years, *p* = 0.886), sex distribution (80% vs. 60% female, *p* = 0.197), or body mass index (20.3 ± 2.3 vs. 21.5 ± 3.0 kg/m^2^, *p* = 0.498), thereby minimizing potential confounding bias in subsequent proteomic analyses. Hemodynamic evaluation revealed characteristic abnormalities in the IPAH cohort, including significantly elevated mPAP (40.2 ± 6.2 mmHg) with normal PCWP (8.1 ± 2.8 mmHg). The patients demonstrated impaired cardiac function, as evidenced by reduced cardiac output (2.9 ± 1.2 L/min) and cardiac index (2.1 ± 0.9 L/min/m^2^), along with markedly increased PVR (4.5 Wood units). Notably, NT‐proBNP levels were profoundly elevated in IPAH patients (1386.25 ± 618.18 pg/mL) compared with controls (62.15 ± 23.48 pg/mL, *p* < 0.001), reflecting significant right ventricular strain.

### 3.2 Quantitative Proteomic Analysis and Quality Control

**FIGURE 1:**
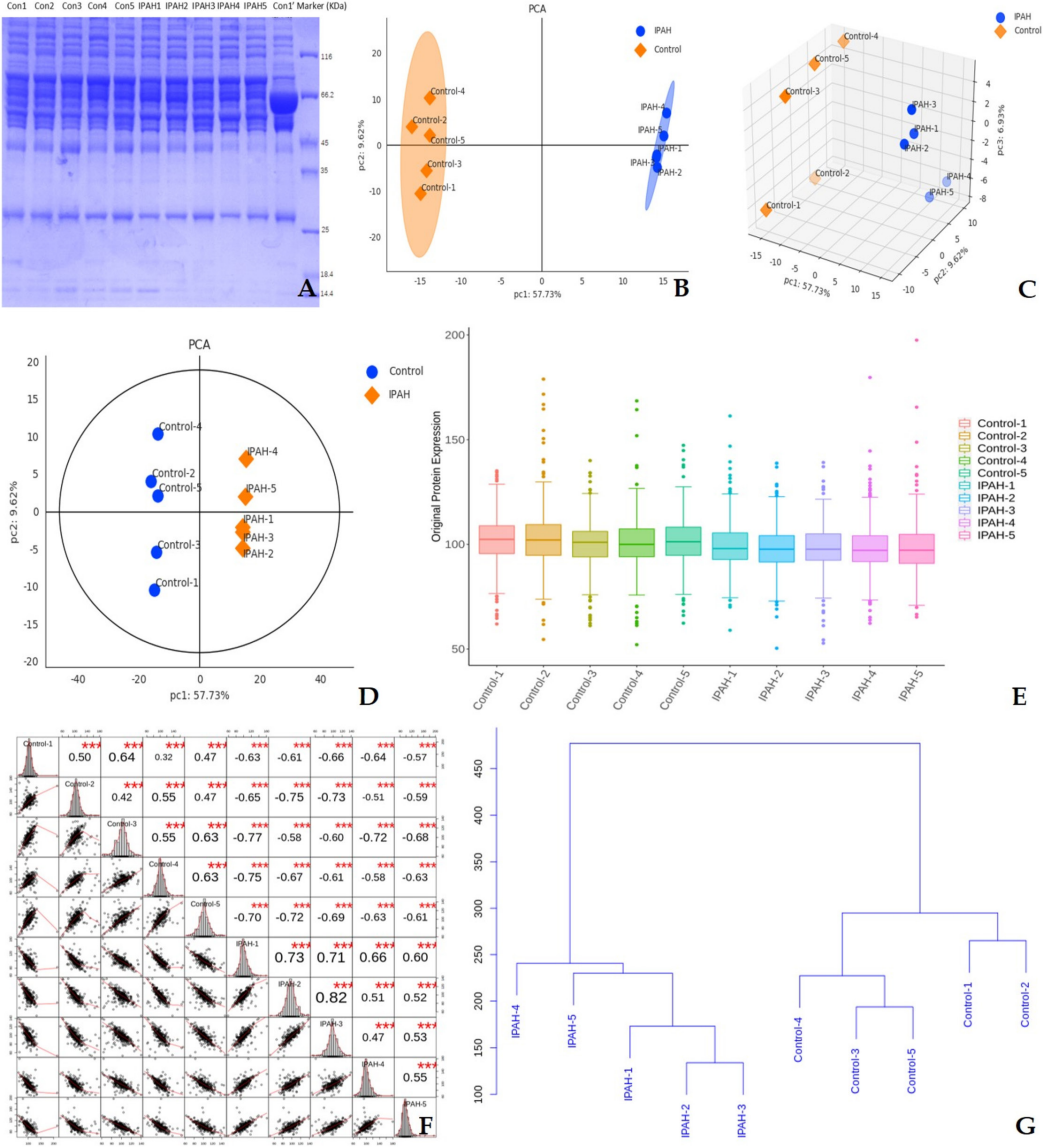
Data quality control analysis based on IPAH and control serum specimens. (A) SDS‐PAGE electropherograms based on IPAH and control serum specimens. Principal component analysis (B–D), visual display (E), correlation analysis (F), and hierarchical clustering dendrogram (G).

Our quantitative proteomic analysis identified a total of 401 unique proteins and 1554 distinct peptide sequences through liquid chromatography–tandem mass spectrometry analysis of IPAH patient and healthy control samples, followed by rigorous database searching and quality control procedures. As illustrated in Figure 1, SDS‐PAGE electropherograms (Figure 1A) initially characterized protein composition differences, and principal component analysis (Figure 1B–D) revealed distinct clustering patterns, clearly separating IPAH and control samples along principal components, with PC1 and PC2 explaining 57.7% and 9.6% of variance, respectively. Visual display of original protein expression (Figure 1E) enabled intuitive comparison of expression levels across samples. Correlation analysis (Figure 1F) quantified sample‐to‐sample expression similarities, while hierarchical clustering (Figure 1G) further grouped samples based on proteomic profiles, consistently differentiating IPAH from controls. These results confirmed reliable data quality and significant proteomic divergence between the two groups.

### 3.3 Differential Protein Expression

**FIGURE 2:**
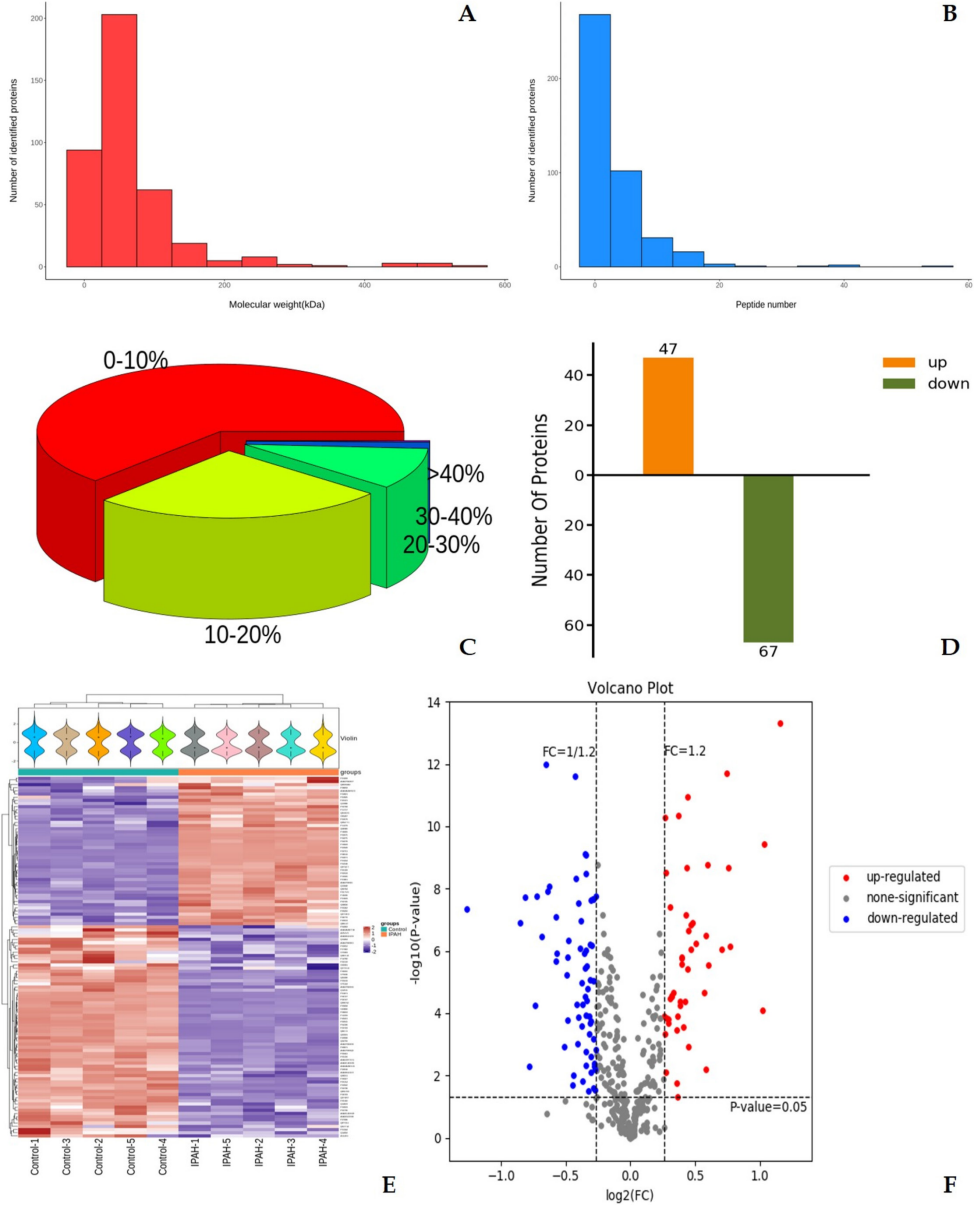
Basic qualitative statistics of original protein expression based on raw mass spectrometry data. (A) Number of identified proteins corresponding to different molecular masses. (B) Number of identified proteins corresponding to different peptide number. (C) Coverage of peptide sequence relative to complete protein sequence. (D) Differential protein graph. (E) Clustering heat map of differential protein expression levels. (F) Differential protein volcanic map.

Basic qualitative statistics of reliable protein expression derived from raw mass spectrometry data were summarized in Figure 2. The majority of identified proteins fall within 10‐ to 120‐kDa lower molecular weight ranges (Figure 2A). The peptide number distribution per protein displayed optimal identification of most proteins (> 90%) with 0–20 peptides, indicating robust protein coverage (Figure 2B). Proteins with 0%–10% peptide sequence coverage relative to their complete amino acid sequences represented the predominant fraction among all identified proteins (Figure 2C). The histogram, volcano plot, and clustering heatmap (Figure 2D–F) reveal 114 DEPs between groups, with 47 up‐regulated and 67 down‐regulated proteins (Tables S2 and S3). Collectively, these analyses characterize initial protein expression features at the raw data level, highlighting differential expression patterns.

### 3.4 Functional Enrichment Analysis

**FIGURE 3:**
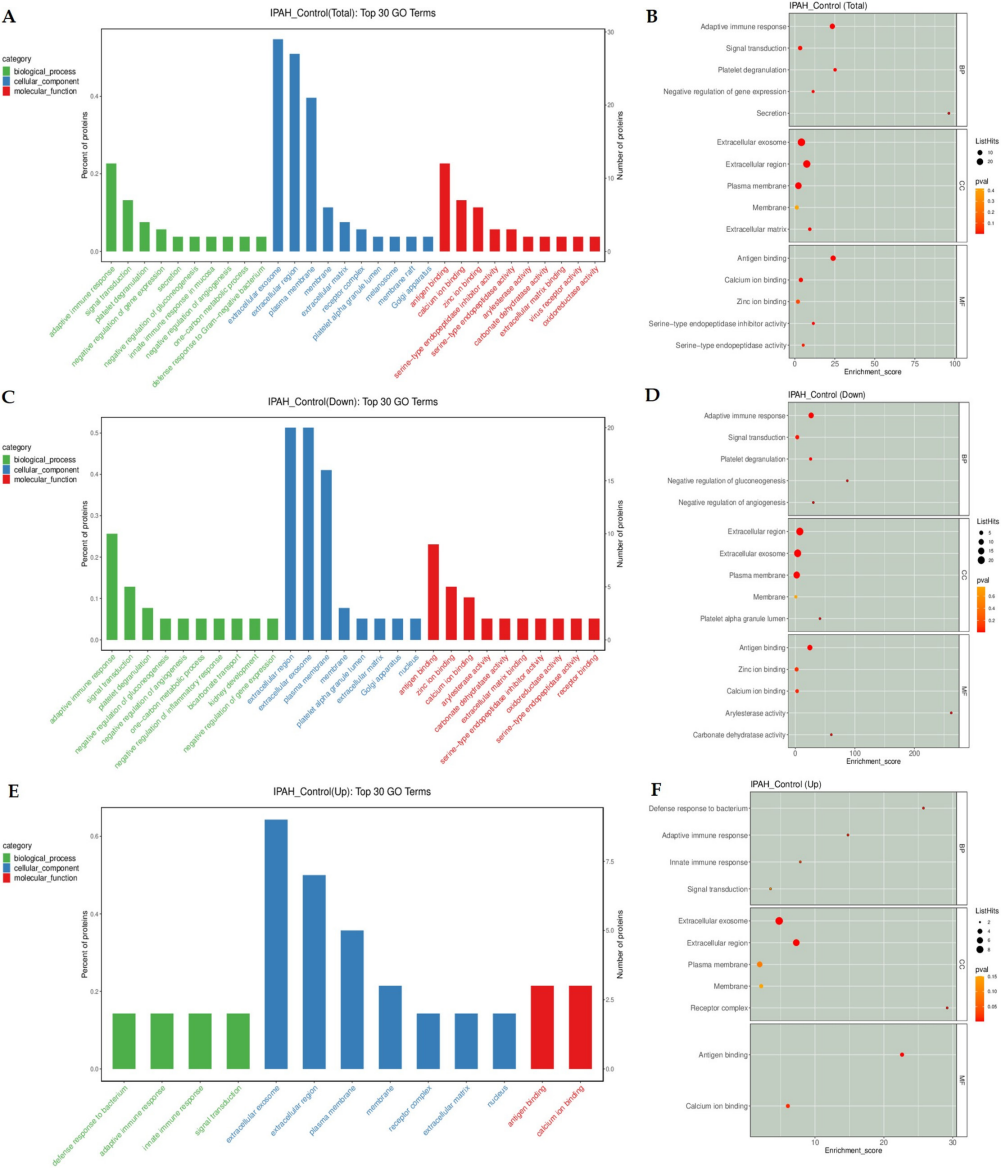
Gene Ontology (GO) enrichment analysis of differential expression protein between IPAH and control group. (A,B) Total top GO terms (IPAH vs. control). (C,D) Down‐regulated IPAH versus control top GO terms. (E,F) Up‐regulated IPAH versus control top GO terms.

GO enrichment analyses stratified by biological process (green), cellular component (blue), and molecular function (red) for IPAH patients and control groups were shown in Figure 3. In biological process, DEPs are primarily involved in adaptive immune response, followed by signal transduction (Figure 3A–F). For molecular function, DEPs are mainly involved in antigen binding and calcium ion binding. Extracellular exosome and extracellular region are the most commonly enriched cell components (Figure 3A–F). The top 30 GO terms of the three aspects in up‐regulated and down‐regulated DEPs between IPAH and controls exhibit similar distributions (Figure 3A–F).

**FIGURE 4:**
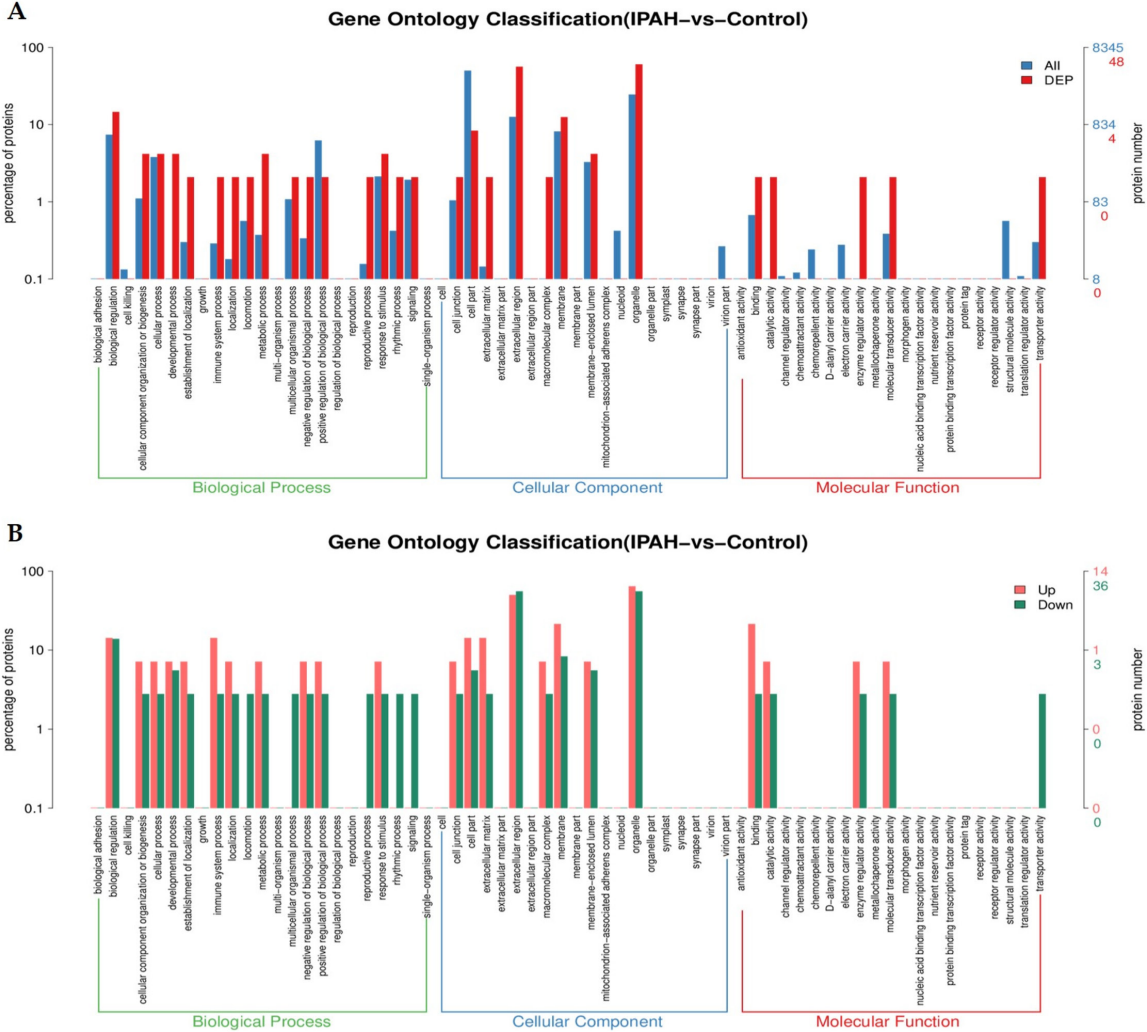
Comparative distribution of differentially expressed proteins and species proteins at Gene Ontology (GO) Level 2. (A) GO classification between IPAH and control group (total). (B) GO classification between IPAH and control group (up‐regulated and down‐regulated).

Comparative distribution of DEPs was further analyzed at GO Level 2, revealing distinct functional characteristics (Figure 4A,B). In biological processes (green), both up‐regulated and down‐regulated proteins were significantly enriched in biological regulation, while up‐regulated proteins showed additional predominant involvement in immune system processes. Regarding cellular components (blue), both categories demonstrated significant association with extracellular region and organelles, with up‐regulated proteins further enriched in membrane. At the molecular function level (red), shared annotations included enzyme regulator activity and molecular transducer activity, whereas up‐regulated proteins specifically exhibited binding capacity, and down‐regulated proteins specifically exhibited transporter activity (Figure 4B).

**FIGURE 5:**
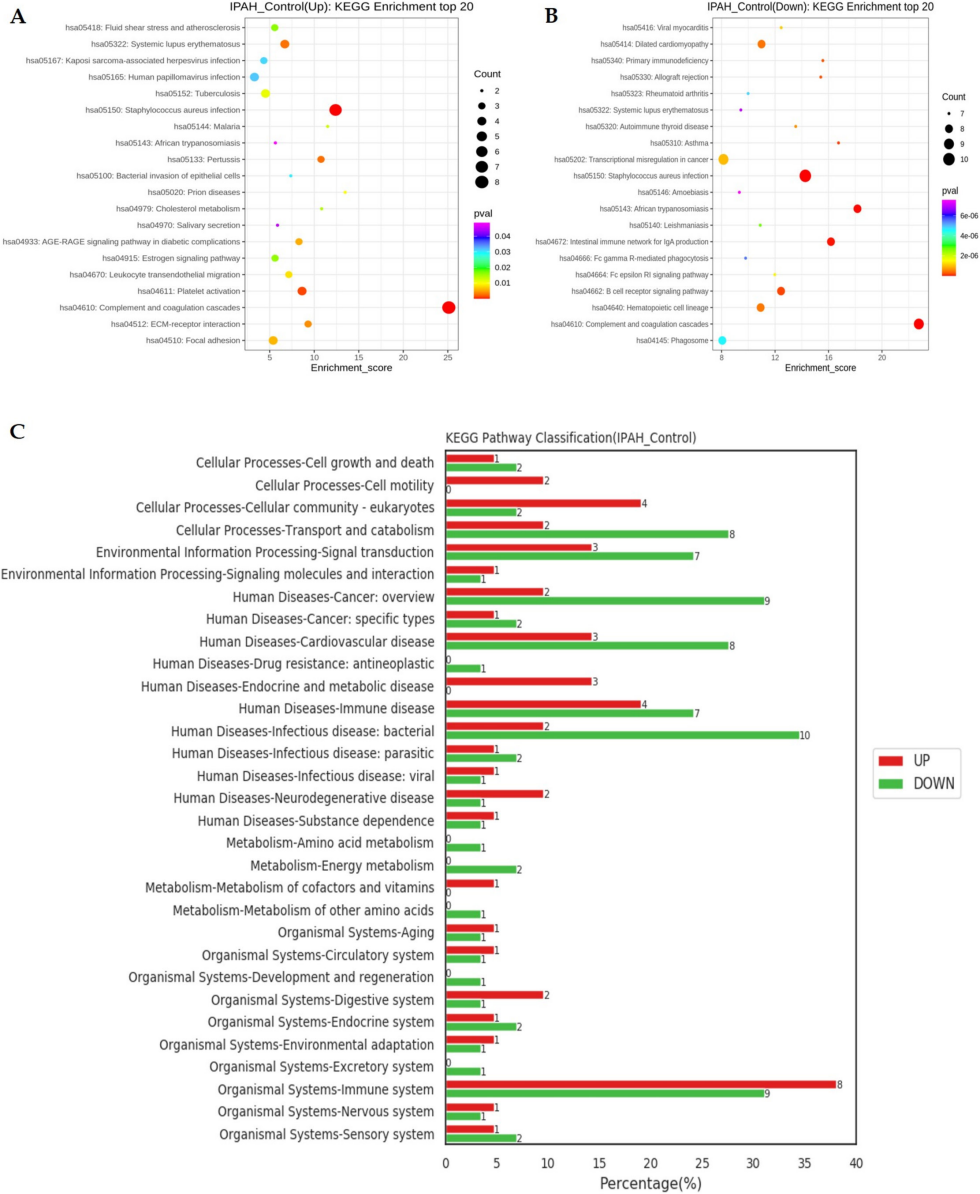
Kyoto Encyclopedia of Genes and Genomes (KEGG) enrichment analysis of differential expression proteins between IPAH and control group. (A) Top 20 KEGG enrichment terms (IPAH vs. control up‐regulated) shown in bubble diagram. (B) Top 20 KEGG enrichment terms (IPAH vs. control down‐regulated). (C) KEGG pathway classification (red, up‐regulated; green, down‐regulated) at KEGG Level 2.

The enrichment analyses of DEPs in the KEGG pathway are depicted in Figure 5. Both up‐regulated and down‐regulated proteins were significantly enriched in complement and coagulation cascades (Figure 5A,B). KEGG Level 2 pathway analysis demonstrated distinct functional distributions between up‐regulated and down‐regulated protein groups (Figure 5C), and the up‐regulated proteins showed predominant enrichment in “Organismal Systems–Immune system.” In contrast, down‐regulated proteins were primarily associated with pathways related to “Human Diseases–Infectious disease: bacterial” and “Human Diseases–Cancer: overview.”

### 3.5 Protein Interaction Network Analysis

**FIGURE 6:**
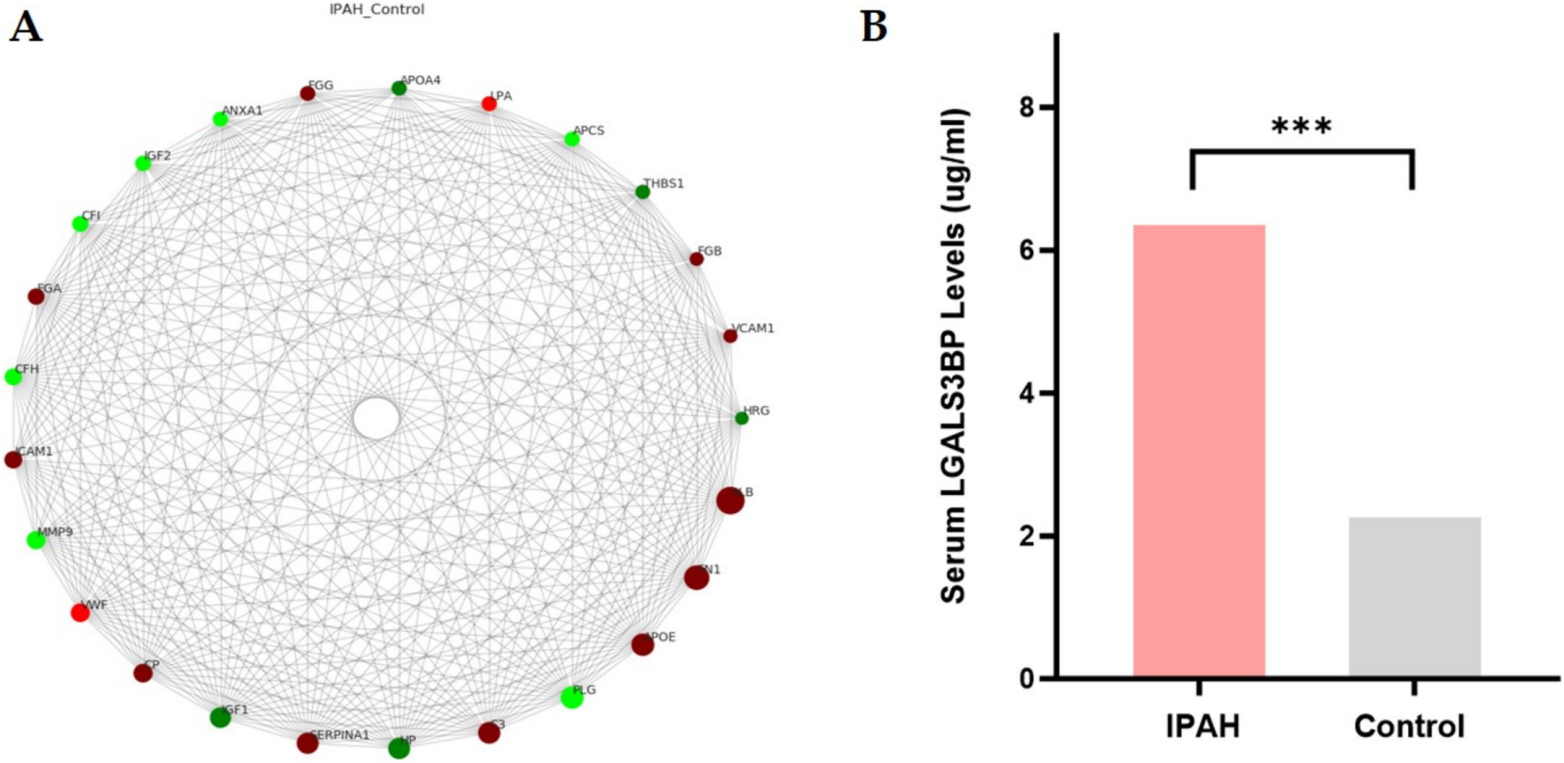
Visualization and validation of potential proteins. (A) Visualization of the top 25 proteins displayed under gene names for node connectivity using the python software package “networkx.” (B) Serum LGALS3BP expression by ELISA analysis between IPAH and control group.

The PPI network among DEPs between IPAH and controls was constructed using the STRING database and illustrated in Figure 6A. Nodes represent proteins (red: up‐regulated in IPAH; green: down‐regulated), and edges denote their interactions, revealing a complex network of dysregulated molecular associations in IPAH. Among these interactions, complement activation (such as complement C3/complement factor H), coagulation cascade, and growth factor regulation (such as insulin‐like growth factor 1/insulin‐like growth factor‐binding protein 1) were highlighted, revealing a network centered around three core physiological systems: immune response, coagulation, and growth factor signaling.

### 3.6 LGALS3BP Validation

While proteomic profiling has revealed multiple DEPs in IPAH, a substantial proportion of these proteins have been well documented in prior studies, such as von Willebrand factor (vWF) [11], intercellular adhesion molecule 1 (ICAM1) [12], and insulin‐like growth factor‐binding protein 2 (IGFBP2) [13]. In pursuit of novel, clinically actionable serum biomarkers for IPAH, we prioritized LGALS3BP for comprehensive functional characterization based on its distinctive expression pattern and potential pathophysiological relevance. Proteomic analysis demonstrated up‐regulation of LGALS3BP in the IPAH group compared with the control group (FC = 1.36, *p* < 0.001). Further GO analysis indicates LGALS3BP is a multifunctional protein primarily involved in extracellular compartments, exosomes, blood microparticles, and proteinaceous extracellular matrix, suggesting roles in intercellular communication and matrix remodeling. Its association with scavenger receptor activity and cellular defense response highlights its involvement in pathogen recognition and immune surveillance, potentially modulating inflammatory responses. Thus, further ELISA analysis was performed to compare LGALS3BP in an expanded validation cohort (*n* = 30 per group). This cohort reproduced and extended the findings from the discovery‐phase cohort, with IPAH patients displaying significant clinical impairments (Table 1). Functional capacity was markedly reduced, as evidenced by substantially diminished 6‐min walk distance (338 vs. 507 m, *p* < 0.001), predominantly NYHA functional class II–IV (90% of patients), and increased NT‐proBNP (2921.5 vs. 74.1 pg/mL, *p* < 0.001), in stark contrast to the entirely asymptomatic control group. Invasive hemodynamics were severely compromised in the IPAH group, evidenced by elevated right atrial pressure, mPAP, and PVR, along with reduced cardiac output and cardiac index. ELISA analysis quantifies serum LGALS3BP levels, showing a significant increase in the IPAH group compared with controls (6.43 ± 1.73 vs. 2.33 ± 1.06 μg/mL; *p* < 0.001, Figure 6B), suggesting its potential as a pathogenic mediator and biomarker in IPAH.

**TABLE 1:** Comparison of baseline characteristics between IPAH patients and healthy controls in the validation cohort.

Parameters	IPAH group (*n* = 30)	Control group (*n* = 30)	*p*
Age (year)	33 (12–72)	56 (20–74)	< 0.001
Female, *n* (%)	23 (76.7)	26 (86.7)	0.458
BMI (kg/m^2^)	20.29 (16.42–31.24)	22.34 (17.15–40.32)	0.392
6MWD (m)	338 (164–398)	507 (423–608)	< 0.001
NYHA‐FC (*n*, %)	I (3, 10%) II (15, 50%) III (8, 26.7%) IV (4, 13.3%)	I (30, 100%)	
NT‐proBNP (pg/mL)	2921.5 (500–7800)	74.1 (37–2923)	< 0.001
Creatinine (μmol/L)	79.00 ± 22.96	22.00 ± 14.91	0.006
Uric acid (μmol/L)	484.00 ± 78.3	301.32 ± 80.21	< 0.001
LGALS3BP (μg/mL)	6.43 ± 1.73	2.33 ± 1.06	< 0.001
mRAP (mmHg)	11 (4–18)		
mPAP (mmHg)	58 (39–87)		
PCWP (mmHg)	8 (3–16)		
PVR (Wood units)	19 (8–29)		
CO (L/min)	2.45 (1.80–2.90)		
CI (L/min/m^2^)	1.56 (1.11–2.11)		

Abbreviations: 6MWD, 6‐min walking distance; BMI, body mass index; CI, cardiac index; CO, cardiac output; IPAH, idiopathic pulmonary arterial hypertension; mPAP, mean pulmonary arterial pressure; mRAP, mean right atrial pressure; NT‐proBNP, N‐terminal brain natriuretic peptide precursor; NYHA‐FC, New York Heart Association Functional classification; PCWP, pulmonary capillary wedge pressure; PVR, pulmonary vascular resistance.

## 4 Discussion

Proteomic profiling has emerged as a powerful tool for biomarker identification, and several targeted proteomic studies have sought to identify novel biomarkers with diagnostic and prognostic potential in PAH. Rhodes et al. [14] identified a prognostic panel of nine proteins, including IL1RL1/ST2, TIMP‐1, TIMP‐2, plasminogen, ApoE, erythropoietin, complement factors H and D, and IGFBP‐1, that provided independent prognostic value beyond NT‐proBNP. These biomarkers collectively reflect multiple pathophysiological pathways in PAH, encompassing myocardial stress, inflammation, pulmonary vascular cell dysfunction, iron metabolism dysregulation, and coagulation abnormalities. In a complementary study, Lavoie et al. [15] performed a comprehensive proteomic analysis of blood endothelial cells from hereditary PAH patients with BMPR2 mutations. Their investigation revealed significant alterations in 22 out of 416 detected proteins, highlighting distinct molecular signatures in the vascular endothelium of genetically predisposed PAH patients.

In this study, TMT‐based quantitative proteomics were used to analyze serum samples from RHC‐confirmed IPAH patients and age‐, sex‐ and BMI‐matched healthy controls, identifying 401 high‐confidence proteins predominantly comprising low‐molecular‐weight species with limited peptide segments, among which 114 DEPs (47 up‐regulated and 67 down‐regulated) were detected, with GO analysis revealing up‐regulated proteins to be significantly enriched in adaptive immune response pathways and associated with extracellular matrix organization, extracellular vesicle function, and antigen binding capacity, while KEGG pathway analysis demonstrated their predominant involvement in infectious disease and immune system regulation pathways. These enriched pathways, such as adaptive immune response, complement/coagulation cascades, and cell adhesion/extracellular matrix organization, are integral to the established paradigm of PAH pathophysiology, which centers on the vicious cycle of inflammatory stimulation and proliferative response [16, 17, 18, 19]. In this context, the adaptive immune and complement cascades sustain a pro‐inflammatory milieu, which not only initiates and perpetuates endothelial injury but also amplifies perivascular inflammation and thrombosis. These events collectively drive the characteristic phenotypic switching of vascular cells and pathological extracellular matrix remodeling, culminating in vascular wall thickening and stiffening. Notably, our analysis detected significant up‐regulation of several established IPAH‐associated proteins among the DEPs, such as vWF (FC = 2.05, *p* < 0.001), ICAM1 (FC = 1.25, *p* < 0.001), IGFBP2 (FC = 1.69, *p* < 0.001), and fibronectin 1 (FC = 1.68, *p* < 0.001) [11, 12, 13, 20], which is consistent with previous reports that collectively corroborate the involvement of extracellular matrix remodeling and inflammatory pathways in IPAH pathogenesis.

Based on our previous findings, elevated LGALS3BP levels were significantly correlated with hemodynamic impairment, reflected disease severity, and independently predicted mortality in PAH patients [21]. In pursuit of novel, clinically actionable serum biomarkers for IPAH, we prioritized LGALS3BP for further investigation based on its distinct expression pattern and potential clinical relevance. Our proteomic analysis revealed significant up‐regulation of LGALS3BP in IPAH patients compared with healthy controls (FC = 1.36, *p* < 0.001), with subsequent ELISA validation confirming this finding, which provides a theoretical foundation for understanding the pathophysiological relevance of LGALS3BP in IPAH. LGALS3BP is an 80‐ to 100‐kDa secreted glycoprotein belonging to the Group A scavenger receptor cysteine‐rich superfamily [22, 23], expressed broadly in epithelial cells and multiple tissues [24, 25]. Although not previously investigated in PAH, its documented roles in modulating inflammation and cell proliferation align with core pathological processes in pulmonary hypertension. As a multifunctional ligand, LGALS3BP interacts with galectin‐3, a well‐characterized driver of reactive oxygen species, fibrosis, and inflammation in PAH [26]. Previous studies have demonstrated that LGALS3BP, secreted by neuroblastoma cells, binds to galectin‐3 on bone marrow mesenchymal stem cells and activates the Ras/MEK/ERK signaling pathway, up‐regulates three C/EBP‐binding sites on the IL‐6 promoter, ultimately enhancing IL‐6 transcription and secretion [27]. Moreover, LGALS3BP also contributes to hepatic fibrosis and carcinogenesis via regulation of TGF‐β1 signaling [28]. These findings lead us to propose that LGALS3BP may play a significant role in the pathogenesis and progression of IPAH. As a secreted glycoprotein capable of engaging integrins and galectins, LGALS3BP may serve as a molecular bridge linking immune activation to extracellular matrix dysregulation. It potentially amplifies inflammatory responses through leukocyte recruitment and drives vascular remodeling by promoting phenotype transition and smooth muscle cell proliferation. These effects might be mediated through the modulation of key pathogenic molecules in PAH, notably IL‐6 and TGF‐β1 [29, 30], thereby linking the immune‐driven injury to the structural remodeling in IPAH.

While our clinical data reveal a significant association between LGALS3BP levels and disease severity in IPAH, the precise mechanistic roles of this protein in pulmonary vascular remodeling remain to be fully elucidated. Our further experimental study demonstrated that LGALS3BP expression in lung tissues of monocrotaline‐induced PAH rats was significantly elevated to 1.91‐fold of normal controls (*p* = 0.018, unpublished data). These results align with previous reports showing that LGALS3BP expression in compensated and decompensated right ventricles of monocrotaline‐induced PAH rats increased to 1.37‐fold and 1.41‐fold of normal levels, respectively [31]. This consistent up‐regulation pattern in both pulmonary vasculature and right ventricular myocardium strongly suggests that LGALS3BP may play a crucial pathophysiological role in IPAH development. To bridge this gap and build upon our findings, future studies should aim to delineate the direct pathogenic functions of LGALS3BP. Specifically, in vitro investigations could assess its effects on fundamental cellular processes, such as endothelial cell activation, pulmonary artery smooth muscle cell proliferation and migration, and monocyte chemotaxis, while also evaluating its potential involvement in key inflammatory signaling pathways. Furthermore, in vivo studies utilizing established animal models of PAH, wherein LGALS3BP is modulated via genetic or pharmacological approaches, would be invaluable for validating its causal role and therapeutic potential. These proposed experiments will help translate the correlative associations presented here into a deeper mechanistic understanding of LGALS3BP in IPAH pathogenesis, particularly its potential regulation of critical signaling pathways such as IL‐6 and TGF‐β1 that drive vascular remodeling.

This study has several limitations that warrant consideration. First, the relatively small sample size may limit the generalizability of our findings to the broader IPAH population, and the role of LGALS3BP in other types of pulmonary hypertension remains unknown. Second, while we carefully matched the IPAH and control groups for age and BMI during participant recruitment, residual confounding factors may still influence the results. Third, although we implemented rigorous protein analysis protocols and stringent quality control measures, we cannot completely rule out potential technical variations that might affect protein quantification reliability. Last but not least, while the role of LGALS3BP in the pathogenesis of PAH remains unclear and is not the primary focus of this study, it is nevertheless a direction we are currently exploring further. These limitations highlight the need for future studies with larger cohorts and more comprehensive covariate adjustments to validate our findings.

## 5 Conclusions

Proteomic profiling has emerged as a promising approach for biomarker discovery in IPAH. Our integrated bioinformatics analysis and ELISA validation consistently identified serum LGALS3BP as a potential biomarker for IPAH. Nevertheless, these initial observations necessitate large‐scale, multicenter validation studies to definitively establish the clinical utility of LGALS3BP and other potential biomarkers. Importantly, in‐depth mechanistic investigations are critically needed to delineate the pathophysiological role of these biomarkers in PAH development and progression, which may yield significant insights into disease pathogenesis and reveal new therapeutic opportunities.

## Author Contributions

Mingfei Li, Qi Jin, Lihua Guan, and Daxin Zhou designed the study. Mingfei Li and Qi Jin performed the proteomics analysis and drafted the manuscript. Mingfei Li, Qi Jin, Wenzhi Pan, Dan Tian, Peng Wang, Dandan Chen, Yuan Zhang, and Shasha Chen collected the data from patients. Mingfei Li, Wenzhi Pan, Dandan Chen, and Shasha Chen performed echocardiography and RHC. Lihua Guan and Daxin Zhou revised the manuscript and supervised this study. Junbo Ge provided constructive suggestions. All authors have reviewed and given their approval to the final manuscript.

## Ethics Statement

The study was performed by the Declaration of Helsinki (as revised in 2013). The protocol was approved by the Zhongshan Hospital Institutional Ethics Committee.

## Conflicts of Interest

The authors declare no conflicts of interest.

## Supporting information


**Table S1:** Comparison of baseline characteristics between IPAH patients and healthy controls in the discovery‐phase cohort.
**Table S2:** Differentially expressed proteins up‐regulated in idiopathic pulmonary arterial hypertension versus healthy controls.
**Table S3:** Differentially expressed proteins down‐regulated in idiopathic pulmonary arterial hypertension versus healthy controls.

## Data Availability

All datasets for this study are included in the manuscript and the Supporting Information.
