# Supplementary material for: Serum Proteomic Profiling Uncovers LGALS3BP as a Potential Biomarker for Idiopathic Pulmonary Arterial Hypertension
Source: Clin Respir J. 2025 Nov 27;19(12):e70138. doi: 10.1111/crj.70138 (PMC12659930; doi:10.1111/crj.70138)
Supplement: Supplementary file 1 — Table S1: Comparison of baseline characteristics between IPAH patients and healthy controls in the discovery‐phase cohort. Table S2: Differentially expressed proteins up‐regulated in idiopathic pulmonary arterial hypertension versus healthy controls. Table S3: Differentially expressed proteins down‐regulated in idiopathic pulmonary arterial hypertension versus healthy controls. [file CRJ-19-e70138-s001.docx]

**Supplementary Table 1. Comparison of Baseline Characteristics Between IPAH Patients and Healthy Controls in the Discovery-phase Cohort**

| Parameters | IPAH (n = 5) | Control (n = 5) | P value |
| --- | --- | --- | --- |
| Age (year) | 38.9 ± 11.3 | 37.8 ± 12.2 | 0.886 |
| Female, n (%) | 4 (1) | 3 (2) | 0.197 |
| BMI（kg/m^2^） | 20.3 ± 2.3 | 21.5 ± 3.0 | 0.498 |
| mPAP (mmHg) | 40.2 ± 6.2 | - | - |
| PCWP (mmHg) | 8.1 ± 2.8 | - | - |
| CO (L/min) | 2.9 ± 1.2 | - | - |
| CI (L/min/m^2^) | 2.1 ± 0.9 | - | - |
| PVR (Wood units) | 4.5 ± 0.6 | - | - |
| NT-proBNP (pg/mL) | 1386.25 ± 618.18 | 62.15 ± 23.48 | <0.001 |

IPAH, idiopathic pulmonary arterial hypertension; BMI, body mass index; mPAP, mean pulmonary arterial pressure; PCWP, pulmonary capillary wedge pressure; CO, cardiac output; CI, cardiac index; PVR, pulmonary vascular resistance; NT-proBNP, N-terminal brain natriuretic peptide precursor.

**Supplementary Table 2. Differentially Expressed Proteins Up-regulated in Idiopathic Pulmonary Arterial Hypertension Versus Healthy Controls**

| **Proteins** | ***P* value** | **Fold change** |
| --- | --- | --- |
| Apolipoprotein(a) | 4.76E-14 | 2.228931224 |
| von Willebrand factor | 3.61E-10 | 2.045689918 |
| Fibrinogen-like protein 1 | 8.18E-05 | 2.032443905 |
| Putative uncharacterized protein encoded by LINC00271 | 6.93E-07 | 1.702972973 |
| Insulin-like growth factor-binding protein 2 | 2.06E-09 | 1.689887036 |
| Fibronectin | 2.03E-12 | 1.676210864 |
| Immunoglobulin heavy constant gamma 4 | 8.78E-07 | 1.635740643 |
| Immunoglobulin lambda variable 4-60 | 2.93E-06 | 1.521815889 |
| Sex hormone-binding globulin | 1.71E-09 | 1.514458134 |
| Insulin-like growth factor-binding protein 1 | 0.006444401 | 1.501876407 |
| Vascular cell adhesion protein 1 | 3.14E-07 | 1.501250625 |
| Keratin, type I cytoskeletal 16 | 2.20E-05 | 1.483238143 |
| Fibulin-5 | 5.65E-07 | 1.423067604 |
| Adiponectin | 1.21E-07 | 1.395688623 |
| Protein S100-A8 | 1.46E-07 | 1.38492726 |
| Collectin-10 | 8.62E-07 | 1.381519409 |
| Histone H4 | 0.001202895 | 1.364947966 |
| LGALS3BP | 2.19E-07 | 1.364388744 |
| Fibrinogen alpha chain | 1.14E-11 | 1.356823003 |
| Noelin | 3.68E-06 | 1.356267672 |
| Complement component C7 | 2.13E-09 | 1.351917196 |
| Keratin, type II cytoskeletal 6A | 7.02E-08 | 1.346081652 |
| Immunoglobulin lambda variable 5-52 | 4.33E-05 | 1.343566909 |
| Calmodulin-like protein 5 | 0.00028286 | 1.328754366 |
| Cation-independent mannose-6-phosphate receptor | 1.67E-06 | 1.319953596 |
| Vinculin | 2.70E-06 | 1.319341373 |
| Polymeric immunoglobulin receptor | 1.55E-06 | 1.318877551 |
| Ceruloplasmin | 5.74E-05 | 1.306042435 |
| Serpin A11 | 4.13E-05 | 1.302786093 |
| Fibrinogen beta chain | 4.64E-11 | 1.294036697 |
| Intelectin-1 | 0.047399749 | 1.290721649 |
| Transcriptional repressor NF-X1 | 0.000128955 | 1.288329519 |
| Probable non-functional immunoglobulin kappa variable 3-7 | 0.018140876 | 1.281770477 |
| CD109 antigen | 0.000356688 | 1.281606572 |
| Fibrinogen gamma chain | 2.15E-05 | 1.260112994 |
| Albumin | 2.97E-05 | 1.251238181 |
| Intercellular adhesion molecule 1 | 2.67E-05 | 1.250506415 |
| Keratin, type I cytoskeletal 14 | 3.99E-08 | 1.23914017 |
| Pantetheinase | 3.32E-05 | 1.237136465 |
| Tenascin | 0.000150261 | 1.225906966 |
| Cathelicidin antimicrobial peptide | 0.000206504 | 1.224421708 |
| Cell surface glycoprotein MUC18 | 0.000148626 | 1.2172949 |
| Alpha-1-antitrypsin | 0.007951487 | 1.212610619 |
| Apolipoprotein E | 3.08E-09 | 1.208480565 |
| Complement C3 | 5.26E-11 | 1.206751986 |
| Lysosome-associated membrane glycoprotein 1 | 0.000467792 | 1.204585538 |
| Complement C1q subcomponent subunit A | 0.000129316 | 1.201188642 |

**Supplementary Table 3. Differentially Expressed Proteins Down-regulated in Idiopathic Pulmonary Arterial Hypertension Versus Healthy Controls**

| **Proteins** | ***P* value** | **Fold change** |
| --- | --- | --- |
| Pyruvate dehydrogenase phosphatase regulatory subunit, mitochondrial | 4.61E-08 | 0.417032733 |
| Protein-glutamine gamma-glutamyltransferase 4 | 1.21E-07 | 0.555209953 |
| Netrin receptor UNC5B | 1.87E-08 | 0.570351759 |
| Immunoglobulin kappa variable 2-29 | 0.005191291 | 0.582528881 |
| Insulin-like growth factor I | 5.43E-05 | 0.601537476 |
| Complement factor H-related protein 1 | 1.73E-08 | 0.604781772 |
| Complement factor H-related protein 4 | 3.50E-07 | 0.622485399 |
| Apolipoprotein A-IV | 1.06E-12 | 0.634892086 |
| Carbonic anhydrase 1 | 1.20E-08 | 0.643385374 |
| Histidine-rich glycoprotein | 8.62E-09 | 0.646526177 |
| Haptoglobin | 8.19E-08 | 0.672520488 |
| Selenoprotein P | 2.11E-06 | 0.672520488 |
| Immunoglobulin heavy variable 3-64 | 1.21E-06 | 0.673247449 |
| BPI fold-containing family B member 1 | 0.001170991 | 0.702946687 |
| Thrombospondin-1 | 6.05E-06 | 0.712328767 |
| ATP-dependent RNA helicase A | 0.000170172 | 0.715265866 |
| Hemoglobin subunit delta | 1.61E-06 | 0.715854496 |
| Protein S100-A6 | 4.73E-07 | 0.717917884 |
| Immunoglobulin kappa variable 6D-21 | 0.020958786 | 0.733830414 |
| Arginine-hydroxylase NDUFAF5, mitochondrial | 0.00961686 | 0.738095238 |
| Immunoglobulin heavy constant mu | 2.40E-12 | 0.74263809 |
| Hemoglobin subunit beta | 4.59E-09 | 0.747640685 |
| Immunoglobulin heavy variable 3-49 | 5.39E-05 | 0.749038125 |
| Complement receptor type 2 | 0.000964098 | 0.754693806 |
| Beta-Ala-His dipeptidase | 2.85E-08 | 0.756059009 |
| ICOS ligand | 0.000133599 | 0.756543123 |
| Plasma serine protease inhibitor | 8.54E-07 | 0.763533768 |
| Superoxide dismutase [Cu-Zn] | 1.10E-07 | 0.766961131 |
| Hepatocyte growth factor-like protein | 1.06E-05 | 0.769421341 |
| Calmodulin-like protein 3 | 0.000261998 | 0.769775261 |
| Immunoglobulin heavy variable 2-26 | 5.12E-05 | 0.77444543 |
| ADAMTS-like protein 4 | 0.014615327 | 0.774937877 |
| Pigment epithelium-derived factor | 1.16E-06 | 0.779814881 |
| Immunoglobulin lambda variable 5-39 | 2.91E-05 | 0.783624688 |
| Immunoglobulin heavy variable 5-10-1 | 3.64E-06 | 0.78412132 |
| Insulin-like growth factor-binding protein complex acid labile subunit | 7.63E-10 | 0.785076758 |
| Serum amyloid P-component | 3.30E-09 | 0.786709539 |
| Complement factor I | 9.78E-07 | 0.788128017 |
| Indian hedgehog protein | 0.000117674 | 0.788231086 |
| Plasminogen | 8.12E-10 | 0.788409945 |
| Complement C1q tumor necrosis factor-related protein 3 | 0.001684437 | 0.789549034 |
| WD repeat-containing protein 1 | 0.004691524 | 0.789906943 |
| SPARC-like protein 1 | 4.01E-05 | 0.79147259 |
| Ficolin-3 | 3.02E-06 | 0.792256677 |
| Immunoglobulin kappa variable 2D-30 | 1.63E-05 | 0.794365692 |
| Soluble scavenger receptor cysteine-rich domain-containing protein SSC5D | 0.030417723 | 0.797447879 |
| Carbonic anhydrase 2 | 0.000122452 | 0.802631579 |
| Immunoglobulin lambda variable 3-9 | 0.00047645 | 0.802921024 |
| IgGFc-binding protein | 6.19E-07 | 0.806358382 |
| Apolipoprotein A-II | 0.0002024 | 0.80704607 |
| Probable non-functional immunoglobulin heavy variable 3-35 | 8.53E-06 | 0.807810523 |
| Immunoglobulin heavy variable 3-43 | 0.002437347 | 0.808645325 |
| Insulin-like growth factor II | 0.007623937 | 0.809773756 |
| Complement factor H | 2.40E-08 | 0.810135747 |
| Band 3 anion transport protein | 0.000184446 | 0.810463432 |
| Hepatocyte growth factor activator | 6.71E-07 | 0.812216784 |
| CD5 antigen-like | 2.12E-08 | 0.818661331 |
| Peroxiredoxin-2 | 0.024510988 | 0.819141194 |
| Coagulation factor XIII B chain | 8.88E-06 | 0.820167455 |
| Immunoglobulin heavy variable 1-3 | 0.000678876 | 0.820979785 |
| Immunoglobulin lambda variable 4-69 | 2.16E-08 | 0.821857923 |
| Annexin A1 | 0.00514668 | 0.823969354 |
| Matrix metalloproteinase-9 | 0.004117671 | 0.824001459 |
| Carcinoembryonic antigen-related cell adhesion molecule 7 | 0.029600131 | 0.826484018 |
| Sulfhydryl oxidase 1 | 0.00615379 | 0.827819411 |
| Ubiquitin-conjugating enzyme E2 variant 1 | 0.001460168 | 0.830313015 |
| Coagulation factor V | 1.70E-08 | 0.83266129 |
